# Use of a New Silver-Impregnated Mesh for Incisional Hernia Surgery With Clean-Contaminated Wounds. First Case Series Results

**DOI:** 10.3389/jaws.2025.14786

**Published:** 2025-07-29

**Authors:** Carles Olona Casas, Aleidis Caro-Tarrago, Raquel Casanova, Marc Vallve-Bernal, Cristina Farres, Joan Ferreres, Rosa Jorba

**Affiliations:** Department of General and Digestive Surgery, Joan XXIII University Hospital of Tarragona, Research Group in General and Digestive Surgery (RECERGAD), Tarragona, Spain

**Keywords:** silver-impregnated mesh, contaminated wounds, incisional hernia, surgical site infection, case series

## Abstract

**Aim:**

The incidence of surgical site infection (SSI) in incisional hernia surgery may vary from 10% to 33% in clean-contaminated fields. Although wide-pore polypropylene prostheses are described as being able to resist infection, they are not exempt from morbidity that can lead to a catastrophic scenario associated with high recurrence. To avoid these complications, there are new polypropylene prostheses embedded with silver ions with bactericidal effects. We present the first experience described with the use of this type of prosthesis in a case series of incisional hernia surgery in clean-contaminated fields.

**Material and Methods:**

Single-center, retrospective, observational study on a prospectively collected sample of patients undergoing incisional hernia surgery in clean-contaminated fields. All patients who required ostomy reconstruction or bowel resection and had incisional hernia treated with silver-impregnated prostheses were included. Both procedures were performed in a single procedure using a silver ion-impregnated polypropylene mesh for wall surgery. Demographic data, hernia characteristics, surgical technique and follow-up data are collected. The results obtained in our series are described and compared with a similar previous series of our team using conventional polypropylene prostheses.

**Results:**

From July 2022 to December 2024, 12 patients underwent surgery with clean-contaminated wounds subjected to midline incisional hernia surgery with Optilene Silver Mesh Elastic. Ten retromuscular repairs, one anterior component separation and one onlay repair were performed. The mean follow-up was 12 months, with no SSI or prosthesis explants. At follow-up, the control group presented SSI in 3 (27.3%) cases, compared to 0 cases in the silver mesh group, with differences close to statistical significance (p = 0.052). The rest of the follow-up variables did not show statistically significant differences.

**Conclusions:**

The use of silver-impregnated polypropylene prostheses can be a safe alternative for use in clean-contaminated fields, with no SSI in our series.

## Introduction

In incisional hernia surgery, the incidence of SSI can reach 33% [1], especially in the case of contaminated surgery classified in groups II and III of the United States Centers for Disease Control and Prevention (CDC) [2]. Such contamination can occur in hernia surgeries with concomitant gastrointestinal procedures, inadvertent enterotomy or enterocutaneous fistulas. In these cases the risk of SSI has caused surgeons to avoid or substitute the placement of synthetic meshes. According to the recent literature, the use of macroporous and low weight polypropylene meshes can resist wound infection, reducing the need for explantation of the prosthesis [3–5].

Silver has antibacterial properties and acts upon biofilms; as a result, medical devices impregnated with silver ions are increasingly described [6–8]. Silver exerts prolonged antimicrobial action against grampositive and gramnegative bacteria, fungal species, protozoa and some viruses [2, 9], with no local or systemic toxic effects [10].

A polypropylene mesh impregnated with silver ions has recently been developed. Optilene Silver Mesh Elastic and Optilene Silver Mesh LP (B. Braun Surgical, S.A.) are low weight polypropylene monofilament meshes (48 and 36 g/m^2^, respectively) of large pore size (3.2 and 1 mm, respectively) impregnated with silver ions in the form of zeolites. They are indicated for inguinal, ventral and incisional hernia repair and for the prevention of incisional hernia in patients at risk, such as those with concomitant gastrointestinal surgeries with repair of the abdominal wall or surgeries with a risk of infection. Despite the properties reported in preliminary studies [11–14] data on their efficacy in incisional hernia surgery involving clean-contaminated fields are lacking. The present study evaluates the first results obtained in terms of SSI, morbidity and recurrence using the Optilene Silver Mesh in hernia surgery with clean-contaminated wounds.

## Materials and Methods

### Study Design and Population

A retrospective, case series single-center study was made involving a prospectively recruited sample of patients subjected to incisional hernia surgery with clean-contaminated wounds.

The study is carried out in a University Hospital, reference center in the province for colorectal and complex abdominal wall surgery. In 2019 we established a team made up of surgeons specialized in colorectal surgery and surgeons with extensive experience in abdominal wall techniques, for the individualized treatment of patients who require ostomy reversal and abdominal wall reconstruction [15].

All patients subjected to midline incisional hernia surgery with the Optilene Silver Mesh Elastic were included in the study. All the cases are prospectively recorded in the Spanish Registry of Abdominal Wall Surgery (Registro Español de Cirugía de la Pared Abdominal [EVEREG]), and the protocol was approved by the local Ethics Committee, with previously obtained patient consent.

Each case was evaluated jointly by colorectal surgeons and abdominal wall surgeons, who choose the specific surgical technique based on the conditions and requirements of each patient. Patients were informed of the decided procedures and their possible complications and signed the corresponding informed consent.

All patients were operated on by the same team of surgeons.

### Study Variables

The baseline characteristics of the patients were recorded, along with the risk factors for SSI including gender, age, body mass index (BMI), history of diabetes mellitus, arterial hypertension, chronic obstructive pulmonary disease (COPD), malignancies or inflammatory bowel disease, smoking, previous hernia surgery, and the American Society of Anesthesiologists (ASA) score.

The characteristics of the hernia, such as diameters, reducibility, association with parastomal hernia, recurrence and the need for prehabilitation with botulinum toxin were also documented.

Parameters referred to surgery, such as its elective nature, surgery time, CDC classification of wound contamination, association with other surgeries, surgical technique, mesh size and position, and closure of the fascial defect were recorded.

Follow-up data were registered at discharge, after 30 days and after 12 months in relation to morbidities and type, the presence of surgical wound infection, need for readmission and reoperation, and need for mesh explantation.

A comparative study was conducted involving this new Silver mesh series of 12 cases, which were analyzed and compared to a previously documented series of 11 cases sharing the same clean-contaminated classification, clinical and demographic characteristics using polypropylene prostheses [15]. The objective was to identify potential differences in outcomes. Both series were matched based on relevant variables such as age, gender, hernia and operative characteristics.

### Surgical Technique

Open surgery was performed in all cases, with prior antibiotic prophylaxis using cefuroxime 1 g and metronidazole 500 mg via the intravenous route. Mechanical preparation of the colon 24 h before admission was performed in those patients in which concomitant intestinal surgery was considered.

The technique of choice for the repair of the incisional hernia was retromuscular, along with posterior component separation (Transversus Abdominis Release, TAR) in hernias with a transverse diameter up to 8 cm or in midline hernias associated to parastomal hernias.

A midline incision was made, with retrorectal dissection from medial to lateral. In cases of parastomal hernia without associated midline incisional hernia, the technique used was retromuscular without the need for TAR.

In cases with transverse diameter greater than 10 cm or with impossibility of retromuscular repair, an anterior components separation was performed.

In those cases where simultaneous reconstruction of the intestine was made, the ostomy was disconnected from the abdominal wall and anastomosis was performed prior to incisional hernia repair.

The posterior fascia was closed at midline level and the ostomy orifice (if applicable) using long-term absorbable monofilament running suture [poly-4-hydroxybutyrate (Monomax, B. Braun, S.A.) or polydioxanone (Monoplus, B. Braun, S.A.)] 2-0, employing the short-stitch technique. The mesh was placed in the retromuscular plane with a minimum superposition of 5 cm for all the incisions, fixed with the same suture and cyanoacrylate (Glubran2, GEM).

The anterior fascia in turn was sutured using the same short-stitch technique with poly-4-hydroxybutyrate suture.

In two cases mesh onlay placement was carried out: one patient in which the retromuscular technique could not be used and anterior separation of the component proved necessary, and another patient requiring urgent surgery.

### Statistical Analysis

Data were analyzed using the Statistical Package for Social Scineces (SPSS) version 28.0 (IBM Corp., Armonk, NY, United States). Descirptive statistics were used to summarize the data, including means and standard deviations for continuous variables, and frequencies and percentages for categorical variables.

Comparative analyses between groups were performed using the Chi-square test or Fischer’s exacte test for categorical variables, and the Student’s test for continous variables. A p-value of less than 0.005 was considered statistically significant.

## Results

From July 2022 to December 2024, 12 patients underwent surgery with clean-contaminated wounds subjected to midline incisional hernia surgery with Optilene Silver Mesh Elastic.

The demographic characteristics of the participants are reported in Table 1. With regard to the main risk factors for SSI, the mean BMI was 30.2 kg/m^2^, 5 (41.7%) were active smokers, and 3 (25%) had undergone previous abdominal wall surgery. Patients with colon neoplasia were treated with perioperative chemotherapy and in rectal cancer with radio and chemotherapy. The majority of the patients (66.6%) were classified as ASA III (serious systemic disease).

**TABLE 1:** Demographic characteristics.

Characteristics	Silver N = 12	Control N = 11	p
Gender [n (%)] Male Female	6 (50%) 6 (50%)	5 (45.5%) 6 (54.5%)	0.827
Age [mean (SD)]	60.3 (10.8)	66.5 (12.1)	0.209
BMI (Kg/m^2^) [mean (SD)]	30.2 (4.1)	29.9 (4.03)	0.840
Diabetes Mellitus; yes 8n (%)	1 (8.3%)	3 (27.3%)	0.231
COPD; yes [n (%)]	2 (16.7%)	0 (0%)	0.156
Malignant neoplasm, yes	8 (66.6%)		
Inflamatory bowel disease; yes	1 (8.3%)		
Active smoker; yes [n (%)]	5 (41.7%)	4 (36.4%)	0.795
Prior hernia surgery; yes [n (%)]	3 (25%)	1 (9.1%)	0.315

Most of the operated hernias were classified as corresponding to W2 or W3, with a mean transverse diameter of 7.2 cm, according to the classification of the European Hernia Society [16]. The mean longitudinal diameter was 11.8 cm. A total of 8 (66.7%) of the cases were associated with another parastomal hernia (colostomy 6 cases and ileostomy 2 cases). In two cases the incisional hernia was classified as recurrent, and in another two cases prehabilitation with botulinum toxin was required due to the transverse size of the hernia (Table 2).

**TABLE 2:** Incisional Hernia characteristics.

Characteristics	Silver N = 12	Control N = 11	p
Hernia width	7.25 (4.6)	11.3 (4.7)	0.044
Hernia length [mean (SD)]	11.8 (6.0)	17.5 (10.9)	0.126
Parastomal hernia [n (%)] No Ileostomy Colostomy Colonic fistula	3 (25%) 2 (16.7%) 6 (50%) 1 (8.3%)	2 (18.2%) 2 (18.2%) 7 (63.6%) 0 (0%)	0.744
Recurrent; yes [n (%)]	2 (16.7%)	0 (0%)	0.156
Botulinum Toxin; yes [n (%)]	2 (16.7%)	5 (45.5%)	0.134

Most of the operations were elective (83.3%), while in two cases urgent surgery was indicated. All of the cases were classified as clean-contaminated surgery (CDC group II) due to the presence of ostomies, association to intestinal procedures with mechanical preparation of the colon, or wound contamination. Complete closure of the hernial defect was achieved in all of the patients, with use of the retromuscular technique in 10 (83.3%) of the cases. The mean mesh size was 19.3 × 22.2 cm. Intestinal tract reconstruction was included in three patients (25%) (Table 3).

**TABLE 3:** Operative characteristics.

Characteristics	Silver N = 12	Control N = 11	p
Wound classification CDC II	12 (100%)	11 (100%)	1
Concomitant procedure No [n (%)] Small intestinal Colon Panniculectomy Adhesiolisis	6 (50%) 2 (16.6%) 2 (16.6%) 1 (8.3%) 1 (8.3%)	2 (18.5%)	0.110
Component Separation [n (%)] No Anterior Posterior	9 (75%) 1 (8.3%) 3 (16.7%)	6 (54.5%) 3 (27.3%) 2 (18.2%)	0.459
Mesh width (cm) [mean (SD)]	19,3 (7.9)	23.9 (8.89)	0.271
Mesh length (cm) [mean (SD)]	22.2 (7.3)	26.4 (8.39)	0.240
Mesh position [n (%)] Onlay Retromuscular medial Retromuscular lateral Preperitoneal	2 (16.7%) 8 (66.7%) 2 (16.7%) 0 (0%)	2 (18.2%) 7 (63.6%) 1 (9.1%) 1 (9.1%)	0.715

There were no intraoperative complications. The mean duration of stay was 6 days (range 1–14 days). During admission, one patient suffered surgical wound bleeding that did not require reoperation or drainage. The 30-day control showed one case of subclinical seroma of the surgical wound (Table 4). No surgical wound infections were detected.

**TABLE 4:** Follow-up morbidity.

Follow-up	Silver N = 12	Control N = 11	p
Length of stay (days) [mean (SD)]	6 (3.5)	11.5 (17.5)	0.474
Morbidity Complications; yes (n %) Seroma Bleeding	5 (41.7%) 4 (33.3%) 1 (8.3%)	3 (27.3%) 0 (0%) 1 (9.1%)	0.469 0.093 0.949
Infection SSI [n (%)] No Yes	12 (100%) 0 (0%)	8 (72.7%) 3 (27.3%)	0.052
Reoperation [n (%)]	1 (8.3%)	1 (9.1%)	0.949
Mesh removal	0 (0%)	1 (9.1%)	0.286

Over long-term follow-up (mean 12 months; range 4–24 months) there were three asymptomatic seromas and one recurrence (8.3%) currently pending reoperation. There have been no cases of SSI, and mesh explantation was not necessary in any patient (Table 4).

In the comparative study with our previous series of cases with similar characteristics, all classified as clean-contaminated surgery, no statistically significant differences were found between groups with respect to demographic, hernia and operative characteristics.

At follow-up, the control group presented SSI in 3 (27.3%) cases, compared to 0 cases in the silver mesh group, with differences close to statistical significance (p = 0.052). The rest of the follow-up variables did not show statistically significant differences.

## Discussion

This retrospective study based on a prospective registry found that the use of a polypropylene mesh impregnated with silver ions allows us to treat incisional hernias with clean-contaminated wounds reducing postoperative SSI. These results are particularly significant on considering the dimensions of the treated hernias (W2 or W3 in most cases), their complexity, and the association of parastomal hernias in 41% of the cases.

The association of intestinal surgery or parastomal hernias to midline incisional hernias has been reported to result in SSI in up to 30% of the cases [1]. In the analysis of the Spanish EVEREG incisional hernia registry, cases of simultaneous incisional hernia repair and colorectal surgery account for 55.4% of SSI [17]. The decision is therefore often made to carry out the procedures separately, or even not to operate upon the patient, due to the strong impact upon quality of life. Although it has recently been reported that ventral hernia surgery in contaminated fields can be carried out safely with the use of synthetic macroporous monofilament implants [18] that moreover offer greater savings versus biological matrices [19], this type of surgery is associated with SSI rates of 15%. In our setting we analyzed simultaneous reconstructive ostomy surgery and incisional hernia surgery using light polypropylene meshes, resulting in an SSI rate of 13% [20], which we have been able to reduce even further with the use of silver-impregnated implants. In this study we have made a comparison with a series of our previously published cases by selecting all those classified as clean-contaminated surgery in order to homogenise the groups. We observe that in the control group we obtain 27.3% SSI, and 0 in the silver-mesh group. These differences are close to statistical significance with a p = 0.052, which could reach significance if a larger series were available.

Bacterial contamination of the prosthesis can lead to the formation of biofilms, which favor antimicrobial resistance [21]. In effect, the combination of polysaccharides, proteins, nucleic acids and lipids creates ideal conditions for the growth of bacteria adhered to these materials [22]. Once the biofilm has become consolidated on the implanted mesh material, chronic infection of the wound develops, with fistulas and sinuses that make it necessary to explant the mesh, requiring repeat surgeries that prolong the illness of the patient, worsening quality of life and increasing the healthcare costs.

Silver ions are used as a coating on medical instruments to prevent bacterial contamination [23]. In this regard, it has been suggested that silver ion impregnation may be effective against a wide range of bacteria and biofilms, since the silver ions inhibit biofilm development by increasing the polysaccharide matrix absorption capacity [24].

Optilene Silver Mesh Elastic and Optilene Silver Mesh LP (B. Braun Surgical, S.A.) are the first available silver ion-impregnated meshes. They are made of low weight polypropylene monofilament material (48 and 36 g/m^2^, respectively) with a large pore size (3.2 and 1 mm, respectively), like the meshes indicated to date for hernia surgery in contaminated or clean-contaminated fields, though silver ion impregnation in the form of zeolites increases their resistance to postoperative infection. There are *in vitro* studies on the antimicrobial effect of silver-impregnated meshes that confirm their biocompatibility [25] and antibacterial activity [26], and which serve to recommend the development of implants of this kind [11, 27]. We thus consider the use of silver-impregnated implants to be indicated in the cases described in the present study. For this reason, we are analysing the first series of cases in which this type of mesh has been implanted because its use in incisional hernia surgery has not previously been analysed. The results show them to be safe, with no added morbidity, and they allow us to perform surgery in clean-contaminated wounds, avoiding complications such as SSI, and without requiring mesh explantation in any of the patients. We believe that the protective effect against infection is not only attributable to silver impregnation. As in all types of surgery, it is very important to strictly maintain the surgical infection control measures, with retromuscular placement of the implant, and using meshes of low weight and large pore size, as they are considered to be the best option for surgeries of this type [14]. The presence of silver ions offers an added benefit that may explain the decrease in our SSI rate from 27.3% to 0%.

Our study has some limitations that should be mentioned. The sample size is small, since this is a single-center study, and we moreover wanted to establish a sample as homogeneous as possible, with all the cases being classified as clean-contaminated surgery. A larger sample size—typically 40 to 60 patients—would be needed to meaningfully support claims of improved outcomes over standard meshes, but we consider that the interest of the article lies in describing the first results obtained with this type of mesh. Many centers do not contemplate this type of surgery for the repair of incisional hernias, though in our center it is carried out with the collaboration of colorectal surgeons if needed, ensuring more precise selection of the patients to be operated upon. The different techniques used may also limit the results of the study. Our first choice is always the retromuscular technique but we must adapt to the pathology presented by each patient. We therefore focused on the classification of wound contamination and the association of the type of prosthesis to consider the results obtained. Another limitation may be the short follow-up in some cases (no less than 4 months), though the mean duration of follow-up was 12 months. We consider that longer follow-up is needed; however, as there are no previous publications on the use of meshes of this kind, we feel that our initial results are of interest. The retrospective nature of the analysis could imply selection bias, though in our case we included all the operated patients with the described characteristics, and data collection was carried out prospectively from the EVEREG registry, which is audited and shows high quality and precision [28] for avoiding this type of bias.

### Conclusions

In the first study involving surgeries of this kind, our results suggest that the Optilene Silver Mesh can be a good and safe option for CDC group II incisional hernia surgery, reducing the risk of SSI, with no SSI in our series.

Larger studies involving longer follow-up periods are needed to validate these preliminary results.

## Data Availability

The raw data supporting the conclusions of this article will be made available by the authors, without undue reservation.
